# Deep Brain Stimulation for the Treatment of Hemichorea: Case Series and Literature Review

**DOI:** 10.5334/tohm.765

**Published:** 2023-06-12

**Authors:** Zihan Masood, Joseph S. Domino, Antonia Gragg, Kim Burchiel, Michael Kinsman, Vibhash D. Sharma

**Affiliations:** 1 Department of Neurosurgery, University of Kansas Medical Center, US; 2 Department of Neurological Surgery, Oregon Health & Science University, US; 3 Department of Neurology, University of Texas Southwestern Medical Center, US

**Keywords:** Hemiballismus, deep brain stimulation, hyperkinetic movement disorder

## Abstract

**Background:**

Hemichorea (HC) and its severe form hemiballismus (HB) are rare movement disorders which can be medically refractory to treatments and may need surgical intervention.

**Case Report:**

We report 3 patients with HC-HB who had meaningful clinical improvement with unilateral deep brain stimulation (DBS) of the globus pallidus interna (GPi). We identified 8 prior cases of HC-HB treated with GPi-DBS, and a majority of these patients experienced significant improvement in their symptoms.

**Discussion:**

GPi-DBS can be considered in medically refractory HC-HB in carefully selected patients. However, data is limited to small case series and further studies are needed.

## Introduction

Hemichorea (HC) is a rare movement disorder characterized by involuntary, nonpatterned movements of the distal limbs, while hemiballismus (HB) describes a more severe, high amplitude proximal movement [1,2]. Hemichorea-hemiballismus (HC-HB) encompasses the spectrum of choreic movements that can co-exist and have similar pathophysiology [1]. HC-HB can develop from lesions in the cortex, subthalamic nucleus, thalamus, caudate, and putamen [1,3]. Stroke is the most common etiology. In most patients, the symptoms self-resolve or improve with or without the use of neuroleptic medications, especially in cases of stroke [3,4,5,6]. However, in some patients with medically refractory HC-HB, both pallidotomy and deep brain stimulation (DBS) of the globus pallidus interna (GPi) have improved symptoms [1,3,7]. In this paper we present three cases of HC-HB treated with GPi-DBS and review the current literature on DBS for HC-HB.

## Case Reports

### Case 1

A 68-year-old right-handed woman with diabetes mellitus type 2 (T2DM) presented with 5 years of left upper extremity involuntary movements. She was previously diagnosed with basal-ganglia hemorrhagic hemichorea and did not have good response to multiple medications including haloperidol, baclofen, and clonazepam. Although her choreic symptoms fluctuated, they remained relatively stable over time.

**Figure 1:**
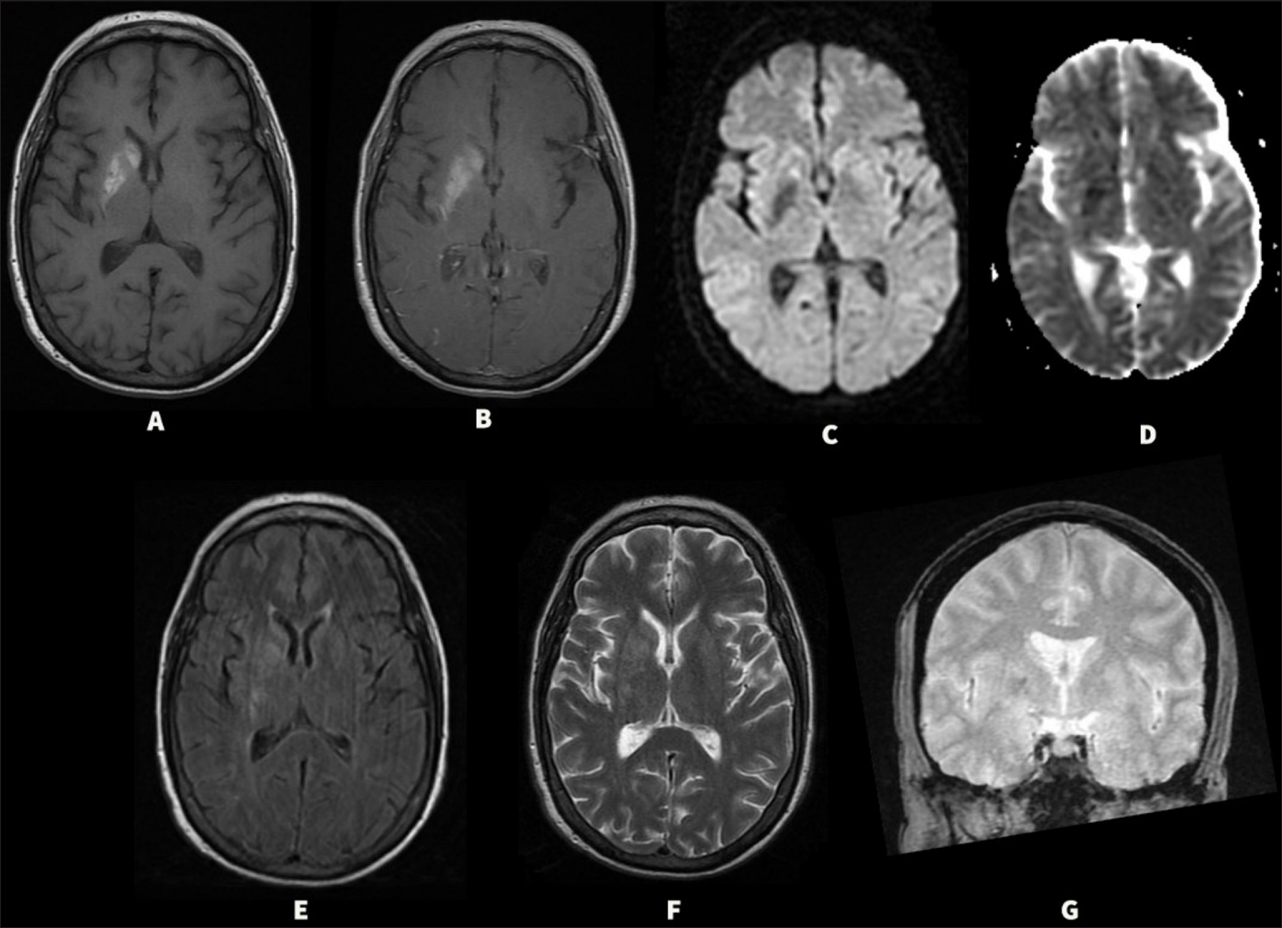
**(Case 1)** Initial MRI showing ill-defined T1 hyperintensities in the right basal ganglia **(A)** without changes in the internal capsule and minimal contrast enhancement with gadolinium without surrounding edema **(B)**. Diffusion weighted sequences showing decreased signal in the right basal ganglia with no restriction **(C & D)**. Fluid attenuated inversion recovery (FLAIR) images show isointense signal **(E)** with hypointenisty on T2 images **(F)** in the basal ganglia. There was no significant gradient echo (GRE) susceptibility to suggest hemorrhage **(G)**.

During our evaluation, she had marked to severe choreic movement with intermittent ballism in left upper extremity (LUE) and moderately so in left lower extremity (LLE). She also had mild choreic movement in the left face. Review of her initial MRI showed an ill-defined lesion in the right caudate and putamen without changes in the internal capsule (Figure 1). There was no gradient echo (GRE) susceptibility to suggest hemorrhage. Review of outside records showed persistently elevated blood glucose in the range of 200–235mg/dL. A repeat MRI brain four years after the initial image did not show any lesions in the basal ganglia. She was diagnosed with Nonketotic hyperglycemia (NKHG) induced chorea.

Due to medication refractory symptoms impairing her quality of life, she was considered a reasonable candidate for DBS therapy and underwent unilateral right GPi-DBS placement without any complications (Activa, lead 3387, Medtronic, Minneapolis, MN, USA). A post-operative CT head confirmed lead placement in the GPi, and the tip of the lead was located at X = +18.09 mm, Y = +2.10 mm, and Z = –4.76 mm relative to the midcommissural point (MCP). With the initial DBS programming, she experienced mild improvement in her symptoms. During the follow-up visits for the next 6 months, she required adjustments to the stimulation, after which she experienced significant functional improvement. At the 1-year follow up, there was moderate to marked improvement in her symptoms corresponding to a score of 2 (much improved) on the Clinical Global Impression-Improvement (CGI-I) scale, and on exam, she had mild to moderate choreic movement in LUE and mild in LLE, and no ballism was present. Although, she required stimulation adjustment over time due to fluctuations in symptoms, her improvement persisted, and at a 5-year follow-up visit, she reported a subjective improvement of about 90%. She was able to return to horse riding. Her DBS programming parameters at her last follow-up visit were C+1–, 3.5 V, 90 PW, 130 Hz.

### Case 2

A 71-year-old right-handed man with coronary artery disease and T2DM presented with 5 months of worsening left sided involuntary movements. He had sudden onset of involuntary movement of the LUE that worsened and involved LLE. He was diagnosed with non-ketotic hyperglycemia (NKHG) induced hemichorea by an outside neurologist and treated with valproate, chlorpromazine, clonazepam, tetrabenazine, and amantadine without significant benefit.

**Figure 2:**
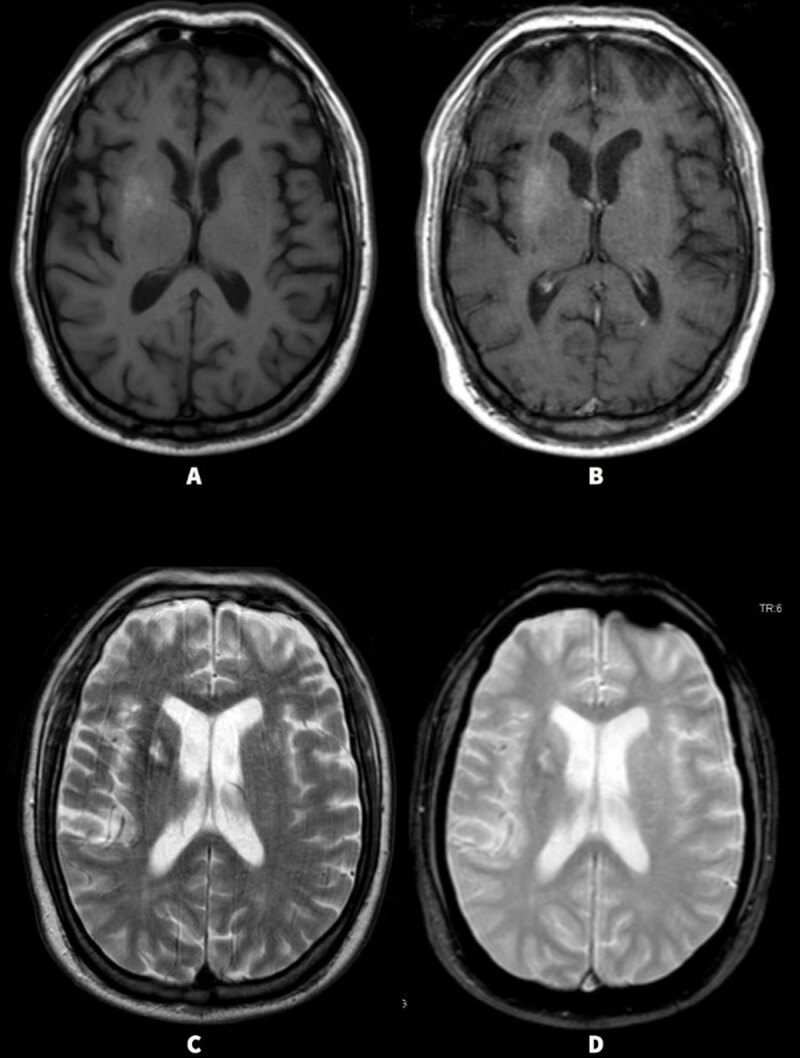
**(Case 2)** Initial MRI brain images showing T1 hyperintensity in the right basal ganglia **(A)** with minimal contrast enhancement **(B)**. In the upper lateral aspects of the right caudate, there was a rim of T2 hypointenisty surrounding a central core of T2 hyperintensity **(C)**. The rim had mixed GRE susceptibility **(D)**. There was no evidence of any edema surrounding the right basal ganglia lesion.

During the evaluation, the patient had HC affecting left upper extremity, left lower extremity and face. The intensity of his choreiform movements varied from moderate to marked, and aggravated with distraction. A review of outside records revealed blood glucoses levels ranging from 61–311 mg/dL, and an MRI brain (Figure 2) showed a lesion in the right globus pallidus and possibly involving the putamen. However, in the upper lateral aspects of the right caudate, there was a rim of T2 hypointenisty with mixed GRE susceptibility, surrounding a central core of T2 hyperintensity (Figure 2). There was no edema surrounding the right basal ganglia lesion.

After an evaluation by the multi-disciplinary team, he was considered a good candidate for right GPi-DBS and underwent surgery (Activa, Lead 3389, Medtronic, Minneapolis, MN, USA) without any complications. Post-operative CT scan confirmed lead placement in the GPi and the tip of the lead was located at coordinates X = +24.69 mm, Y = +4.12 mm, and Z = –2.73 mm relative to the MCP. His symptoms improved with DBS programming and at four months follow-up visit (after two programming adjustments), he reported a 70% improvement in choreic movements. On exam, he has mild choreic symptoms corresponding to a score of 2 (much improved) on a CGI-I scale. His DBS parameters at the last follow-up visit were C+1–, 2.4 V, 60 PW, 130 Hz.

### Case 3

**Figure 3:**
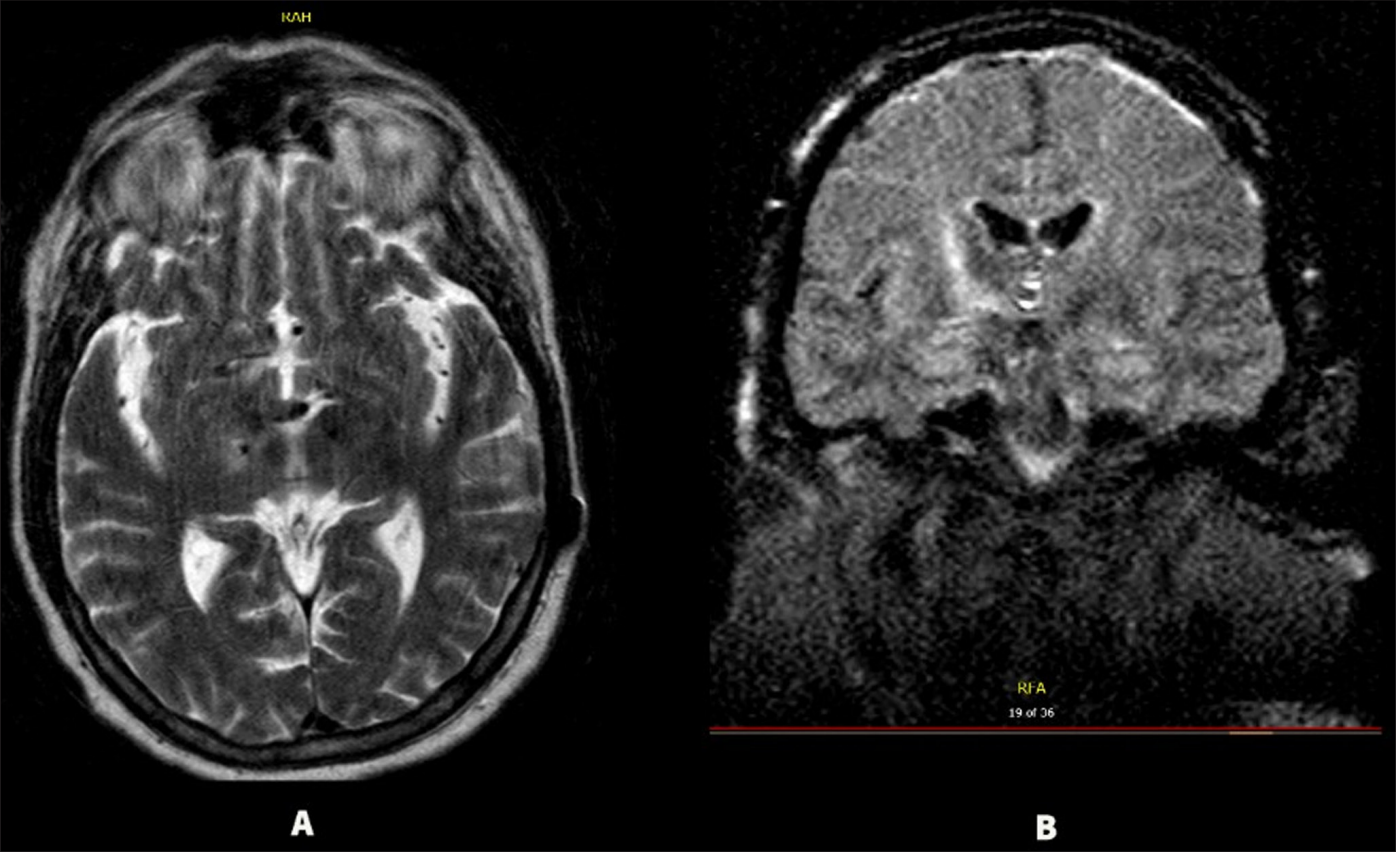
**(Case 3)** MRI brain showing T2 hyperintensity **(A)** and FLAIR changes **(B)** seen around the right electrode compared to his prior imaging with associated restricted diffusion (images not available). The signal abnormalities predominantly involved the right thalamus and basal ganglia.

A 67-year-old man with Parkinson’s disease (PD) underwent bilateral subthalamic (STN) DBS surgery. Four months after surgery, he developed severe, episodic, uncontrolled flailing of the left arm that did not improve with carbidopa/levodopa dose adjustment or changes to the DBS parameters. In fact, turning off the DBS system worsened his symptoms. A repeat MRI brain showed stable lead positions, but revealed T2 hyperintensity and FLAIR changes associated with restricted diffusion around the right electrode in the right thalamus and basal ganglia (Figure 3). Due to concerns about infection, he underwent complete explantation of the right and left DBS system. The culture of the lead grew Propionibacterium acnes, and he was treated with intravenous vancomycin.

A repeat MRI brain four weeks later showed resolution of the previous lesions. However, he continued to have severe chorea in the left arm, albeit with slight improvement. The chorea was significantly bothersome, and he was considered a good candidate for bilateral GPi-DBS. He underwent bilateral GPi-DBS seven months after the initial surgery without any complications. The position of the left lead’s tip relative to MCP was X= –19.02 mm, Y = +2.05, and Z = –5.37 mm, while the position of the right lead’s tip was located at X= +21.05 mm, Y = +2.05, and Z = –4.13 mm. With GPi-DBS, he experienced immediate complete resolution of left HC-HB post-operatively, likely due to microlesion effect. However, after two months, after microlesion benefit dissipated, he had a re-emergence of symptoms and required DBS programming, which resulted in complete resolution of chorea. During last follow-up visit parameters for left Gpi-DBS were C+1–, 4.5V, 60 μs, 120 Hz and right were 8+9–, 4.1V, 60 μs, 120 Hz. Due to capsular side effects, right GP-DBS was programmed in bipolar configuration.

## Literature Review

We performed a PubMed search (key words included were “hemichorea DBS,” “hemiballismus DBS,” “pallidal deep brain stimulation,” “hemichorea-hemiballismus,” “treatment of hemiballismus,” and “treatments for hemichorea”) and found eight other published cases of HC-HB treated with DBS (Table 1) [8,9,10,11,12,13,14,15]. The majority of cases were due to stroke, with other cases resulting from trauma, hyperglycemia, vascular malformation. The duration of symptoms varied from a few weeks to several years. In all the cases, GPi was targeted for the management of medication refractory HC-HB, and the majority of patients had near complete or complete resolution of symptoms with GPi-DBS. However, in our series only one patient experienced complete resolution of symptoms, while the other patients experienced moderate to marked improvement. Some reports of HC-HB after STN-DBS requiring treatment with GPi-DBS have also been published. For example, Pabaney et al. reported a PD patient who developed right HC-HB from peri-electrode hematoma around the left STN after a fall and was subsequently treated with left GPi-DBS, resulting in significant improvement in HC-HB [12]. Similarly, Omaya et al. reported a patient with PD who developed hemiballismus secondary to a stroke adjacent to the right STN-DBS electrode. This patient underwent additional placement of right GPi-DBS which resulted in complete resolution of HC-HB [10]. In another case, Xie et al. reported successful control of HC-HB with GPi-DBS after microhemorrhages from a vascular malformation in the contralateral STN [11].

**Table 1:** Summary of cases.


CASES	AGE (YRS)/GENDER	PRESENTATION	ETIOLOGY	DURATION	TARGET	OUTCOME	DBS PARAMETERS	COMMENTS

Hasegawa et al., 2009	56/M	Left HB	Right subthalamic region hemorrhage	3 years	Right GPi	Resolved at 6 months	C+1–, 4.5 V, 60 μs, 130 Hz	Patient also had mild dystonic posturing of left wrist

Capelle et al., 2011	52/M	Right HC-HB	Post craniopharyngioma resection	3 years	Right GPi + Right VIM	Resolved at initial programming, adjusted at 19 months	3+0–, 0.8 V, 210 μs, 130 Hz	Left VIM-DBS used for long-term stimulation

Omaya et al., 2014	44/M	Left HB	Stroke	1 month	Right GPi	Resolved at 1 week	C+ 1–, 3.3 V, 90 μs, 135 Hz	Stroke adjacent to R electrode for PD, STN electrode remained

Xie et al., 2014	22/M	Left HC	DVA/hemorrhage	4 years	Right GPi	Well suppressed at 10 months	C+1–, 3.6 V, 120 μs, 60 Hz	Microhemorrhage from vascular malformation in right STN

Pabaney et al., 2015	54/M	Right HB	Peri-electrode edema around STN lead	2 weeks	Left GPi	Substantially reduced	C+1–, 2.0 V, 90 μs, 160 Hz	STN-DBS for PD with left STN hematoma after fall.

Son et al., 2017	46/F	Left HC-HB	NKHG	6 months	Right GPi	Resolved at 16 months	2+1–, 3.5 mA, 110–130 μs, 130 Hz	Minimal left calf and foot chorea at 16 weeks.

Ramirez et al., 2018	53/F	Left HB	Stroke	~ 20 years	Right GPi	Near complete resolution at 6 months	C+0–, 3.0 V, 90 μs, 130 Hz	Peripartum infarction in her 30s

Ganapa et al., 2019	46/M	HB	Stroke	Not reported	Right GPi	Improved, lost to follow up	Not reported	

Case 1	68/F	Left HC	NKGH	5 years	Right GPi	Moderate to marked improvement at 1 year	C+ 1–,3.5 V, 90 μs, 130 Hz	Mild fluctuations in symptoms

Case 2	71/M	Left HC	Infarct/NKHG	5 months	Right GPi	Marked improvement at 4 months	C+1–, 2.4 V, 60 μs, 130 Hz	Lost to follow up 1 year post op

Case 3	67/M	Left HB	Infection	4 weeks	Bilateral GPi	Resolved at 2 months	Left: C+1–, 4.5V, 60 μs, 120hz Right: 8+9–, 4.1V, 60 μs, 120hz	Bilateral STN-DBS for PD replaced with bilateral GPi-DBS


* HB-hemiballismus, HC-hemichorea, μs -microseconds, Hz- hertz, DVA – developmental venous anomaly, GPi – Globus pallidus interna, VIM – Ventral intermediate nucleus.

## Discussion

HC-HB are rare movement disorders observed with lesions in the contralateral basal ganglia due to different etiologies [1,3]. Stroke is the most frequent etiology with an incidence of around 0.4–0.5% [6,16]. Common stroke locations include the striatum, followed by the STN, a finding which may be due to the frequency of strokes in these regions [16,17]. Hyperglycemia is another cause of HC-HB, although less frequent compared to strokes. In two series of hyperglycemia-induced HC, all cases showed lesions in the putamen, and about half of the patients had additional basal ganglia lesions [18,19]. The exact pathophysiology of hyperglycemia- induced HC-HB is unclear. Metabolic changes from hyperglycemia causing depletion of GABA and acetylcholine eventually leading to basal ganglia dysfunction has been suggested [20]. Alternatively, imaging findings in NKHG patients suggests ischemic or hemorrhagic changes which lead to basal ganglia dysfunction [18]. Other etiologies, such as neoplasms, demyelinating plaques, infections, drugs, vasculitis, and iatrogenic, are less frequent causes of HC-HB [1,3,4,21,22].

HC-HB from stroke or hyperglycemia usually resolves over time, either by itself or with neuroleptic medications [3,5,6]. In a meta-analysis, around 74% of patients with hyperglycemia-induced HC-HB experienced complete resolution of their symptoms and MRI findings through good glycemic control alone or with a combination of neuroleptics, ranging from 1 day to 10 months, with most resolving within 6 months [18]. In another study of post-stroke HC-HB 73% (11/15) of patients had complete resolution of symptoms, of these 8 patients had resolution in the first 2 months, while the remaining 3 had resolution before the end of first year [5]. Based on the literature, it would be reasonable to trial observation and medical therapies for at least 4–6 months before considering DBS [1,3,4,5,6,18].

Our patient (Case 1) was previously diagnosed with basal ganglia hemorrhage; however, her initial MRI did not show gradient echo (GRE) susceptibility to suggest hemorrhage (Figure 1). Also, the lack of peri-lesional edema with multiple affected regions that are sharply demarcated while preserving anatomic boundaries argued against basal ganglia hemorrhage. Based on these findings and elevated blood glucose levels, a diagnosis of NKHG induced HC-HB was made. A repeat MRI brain four years after the initial MRI did not show any lesions in the basal ganglia or signal on GRE which supports the diagnosis of NKHG induced HC-HB. She had persistence of chorea even after several years, which has been previously reported in a subset of patients with NKHG induced HC-HB [18,19]. In our second patient (Case 2), the initial MRI findings were consistent with NKHG; however, the presence of T2 hypointense rim surrounding a central core of T2 hyperintensity (Figure 2) suggested subacute blood products with necrosis. Given the MRI findings, patient may have had a combination of NKHG and hemorrhagic changes to the right basal ganglia resulting in HC-HB.

GPi-DBS has been shown to significantly improve symptoms in selected patients with medication refractory HC-HB, as shown in Table 1. While other targets such as the Voa and Vop nucleus of the thalamus have been utilized less frequently, Franzini et al. reported improvement in post-traumatic and post-stroke HB with DBS of these targets [23], and Nakano et al. reported rapid resolution of a hyperglycemia induced HC-HB with thalamic stimulation of the Voa/Vop nuclei [24]. In an interesting report by Capella et al., authors implanted DBS leads in both GPi and VIM of the thalamus and found that thalamic target required less stimulation than GPi for complete resolution of symptoms [9]. However, there is more substantial evidence to support pallidal stimulation as the preferred treatment for hyperkinetic movement disorders [25]. The exact mechanism by which GPi-DBS reduces excessive movements is not yet fully understood, but it is suggested that stimulation of the GPi may modulate inhibitory input to the thalamus, thus reducing excessive movements.

Our case series and literature review suggest that GPi-DBS is a potential surgical option for medication refractory HC-HB in carefully selected patients. However, identifying suitable candidates for DBS therapy can be challenging due to the heterogeneity of HC-HB etiologies, and the degree of improvement following DBS therapy is not well defined across different reports. It is unclear why some patients respond better than others. One major limitation of our case series and previously published cases was the lack of standardized objective scales for HC-HB. To aid clinicians in identifying HC-HB patients who may benefit from DBS therapy, additional studies are needed to collect standardized data from multiple centers and include patients with varying HC-HB etiologies.
